# Ethnic differences in prevalence of Dupuytren disease can partly be explained by known genetic risk variants

**DOI:** 10.1038/s41431-019-0483-5

**Published:** 2019-07-30

**Authors:** Sophie A. Riesmeijer, Paul M. N. Werker, Ilja M. Nolte

**Affiliations:** 1 Department of Plastic Surgery, University of Groningen, University Medical Center Groningen, Groningen, The Netherlands; 2 Department of Epidemiology, University of Groningen, University Medical Center Groningen, Groningen, The Netherlands

**Keywords:** Genetic variation, Genetic predisposition to disease

## Abstract

Dupuytren disease (DD), a fibroproliferative disorder of the palmar fascia that causes flexion contractures in the fingers, is prevalent in people of North-Western European descent and less so in other ethnicities. DD is a complex disorder, influenced by genetic risk variants. We aimed to study if the marked differences in prevalences in DD between ethnic (sub)groups could be explained by differences in allele frequencies of the 26 known genetic risk variants of DD. Therefore, genetic risk scores (GRS) composed of the 26 DD risk variants were calculated for the 26 populations from the 1000 Genomes database and correlated to observed DD prevalences from literature. For comparison, GRSs were generated for 10,000 sets of 26 random SNPs and also correlated to the observed DD prevalences to determine the significance of the observed correlation. To determine whether differences in allele frequencies between ethnicities were caused by natural selection, fixation indices (Fst) were calculated from the 26 SNPs and from the sets of 26 random SNPs for comparison. Observed prevalences could be determined from literature for 10 populations. Their correlation with the GRS composed of DD SNPs proved to be 0.60 (*p* = 0.0003). The Fsts between British and other populations were low for European, ad mixed American, and South-Asian populations, and moderate for East-Asians. African populations were significantly different from expected values determined from the random sets. In conclusion, the 26 known genetic risk variants associated with DD explain for a substantial part (*R*^2^ = 0.36) the differing DD prevalences observed between ethnicities.

## Introduction

Dupuytren disease (DD) is a fibroproliferative disorder that causes nodules and cords to form in the palmar fascias. These may extend into the digits and contract, resulting in limited finger extension and function. DD is most often reported in Caucasians of North-Western European descent. [1] The prevalence of DD is high in the UK (8.0–30.0%) [2–7], in Scandinavian countries (3.2–36.0%) [8–10], in the Netherlands (22.1%; >50 years of age) [11], in Flanders (32%; >50 years of age) [12], and in Australia (22%; >60 years of age) [13]. It increases with age and is thought to be associated with hand work [14], diabetes, epilepsy, and liver disease [15]. The number of papers reporting DD in non-Caucasians is increasing, but the reported prevalences are lower than in Caucasians [16, 17]. Saboeiro et al. described a population of over 3 million multi-ethnic individuals including 9938 DD patients [16]. In this population, the prevalence of DD was estimated 0.73% for whites, 0.13% for blacks, 0.24% for Hispanics, 0.07% for Asians, and 0.14% for native Americans. Yeh et al. described a prevalence of 5.65/10^5^ for men and 3.39/10^5^ for women in 1078 Chinese individuals living in Taiwan [18]. A recent study by Lee et al. found a prevalence of 32.2/10^5^ in a large Korean population [19]. Weinstein et al. found a prevalence of 0.53% in Hispanics [20]. Cases of DD in Africans have been described by several authors, but no epidemiological studies have been done. However, DD is thought to be very rare in this ethnicity [21–23]. In 2014, Lanting et al. systematically reviewed the reported DD prevalences of 212 articles and concluded 23 studies had sufficient quality [1]. They found prevalence rates ranging from 0.6% to 31.6% in different population groups and concluded that this spread in prevalence was based on the heterogeneity of the study populations. Hindocha et al. stated that it was not clear whether the extremely variable prevalence of DD in different geographical locations was due to genetic or environmental factors, or a combination of both [17]. However, marked differences in prevalences in combination with the observation of familial clustering point to a genetic component in DD [16].

The heritability of DD, which is estimated to be ~80%, is thought to be due to multiple genes each carrying a small risk [24]. To elucidate this, genome-wide association studies (GWASs) were carried out and so far the largest GWAS identified 26 risk alleles associated with DD [25, 26]. These genetic studies, however, were carried out with Caucasian subjects exclusively. The occurrence and influence of known genetic risk variants on the origin of DD in other ethnicities is unknown. However, variation in risk allele frequencies (RAFs) between populations may account for differences in disease prevalence between populations [27]. Therefore, we aimed to disclose if differences in allele frequencies of 26 known DD susceptibility variants between ethnic groups can explain the ethnic differences in the prevalence of DD.

## Methods

### Study population and genetic risk scores (GRS)

To gain insight in the differences in occurrence of known genetic variants of DD in ethnic groups, we downloaded the genotype data of the 26 DD-associated single nucleotide polymorphisms (SNPs) identified in the recent GWAS [26] from the 1000 Genomes database for 26 populations [28] and compared the RAFs of these SNPs between the populations and between super-populations (Africans, East-Asians, Europeans, Hispanics, and South-Asians). Weighted and unweighted genetic risk scores ((w)GRS) were constructed for each population using the following formulas, respectively:

$${\mathrm{wGRS = }}\frac{{\mathop {\sum}\limits_{{i} = 1}^{n} {{w}_{i}{X}_{i}} }}{{n}} \cdot {\mathrm{GRS}} = \frac{{\mathop {\sum}\limits_{{i = 1}}^{n} {{X}_{i}} }}{{n}}$$

where *i* is the SNP, *n* = 26, i.e. the number of DD-associated SNPs, *w*_*i*_ is the weight for SNP *i* (i.e. the natural log of the odds ratio (OR) for DD of SNP *i* from the DD GWAS), [26] and *X*_*i*_ is the number of risk alleles the person carries of that SNP. Note that the unweighted GRS is just the mean number of risk alleles. The wGRS and GRS were calculated using Plink [29].

### Statistical analyses

#### Prevalences and correlation

We hypothesized that if DD risk alleles occur less in a certain ethnic group, the prevalence of DD in this ethnic group would be lower. To test this hypothesis we correlated (unweighted) population GRSs based on the DD RAFs with observed prevalences of DD in populations using Pearson’s correlation coefficient. Observed prevalences were determined from literature. Our final literature search was performed on February 10, 2018. PubMed was searched with the following search terms: ‘Dupuytren Contracture’ OR ‘Dupuytren disease’ OR ‘Dupuytren’ AND ‘Prevalence’ OR ‘Incidence’. Only studies describing the prevalence in the general (non-comorbid) population were considered for determining the observed DD prevalence in populations. Also, studies describing the prevalence in merely one sex were excluded, as DD occurs more frequently in men [30]. Studies with insufficient sample size (*n* < 100) were also excluded. When available for a population, studies with positively assessed quality were preferred [1]. Study’s age ranges were taken into account as DD prevalence is known to increase with age [1]. For this we determined for each study the age range and calculated the relative contribution of this age group to the prevalence of DD in the Dutch population using the prevalence data of Lanting et al. per age group (decade) (Fig. 2 from Lanting et al. [1]) and age distribution in the general Dutch population from Statistics Netherlands [31]. We here assumed that in each country the prevalence distribution over age is similar to that in the Netherlands. The prevalence estimate from the study was next divided by this relative contribution to provide an estimate of the prevalence of DD in the respective study’s population for all ages. When DD prevalences from multiple studies were available for one population, the prevalence was calculated as the mean of the prevalences of available studies, weighted by study sample sizes.

To determine the significance of the observed correlation between DD SNPs and prevalence (unweighted) population GRSs of 10,000 sets of 26 random SNPs [28] with RAFs similar to those of the DD risk variants in British individuals (<1% difference) [27] were generated using Plink [29] and correlated with observed DD prevalences. The significance was next calculated as the proportion of sets of random SNPs that exhibited a larger correlation than the observed one. A *p*-value < 0.05 was regarded significant. The Pearson’s correlation analyses were performed in R [32] and Microsoft^®^ Excel.

#### Population differentiation

To determine if the differences in DD prevalence can be explained by population differentiation or natural selection, the fixation index (Fst) was calculated with VCF tools (https://vcftools.github.io/index.html). Population Fst values between the British population (GBR) and the other 25 1000 Genomes populations [28] were computed for the 26 known DD SNPs, since these SNPs were identified using a British population. In addition global SNP Fst values were determined as well. The Fst values were also computed for the 10,000 sets of 26 random SNPs mentioned above to assess an empirical distribution. Significances of the observed Fst values were next determined using the 2.5% and 97.5% percentiles of the empirical distribution to assess whether populations were more or less similar to the British GBR population from 1000 Genomes [28] for the 26 DD SNPs than expected and whether SNPs were more or less differentiated than expected. A *p*-value of < 0.05 was regarded as significant.

## Results

### Allele frequencies

**Fig. 1:**
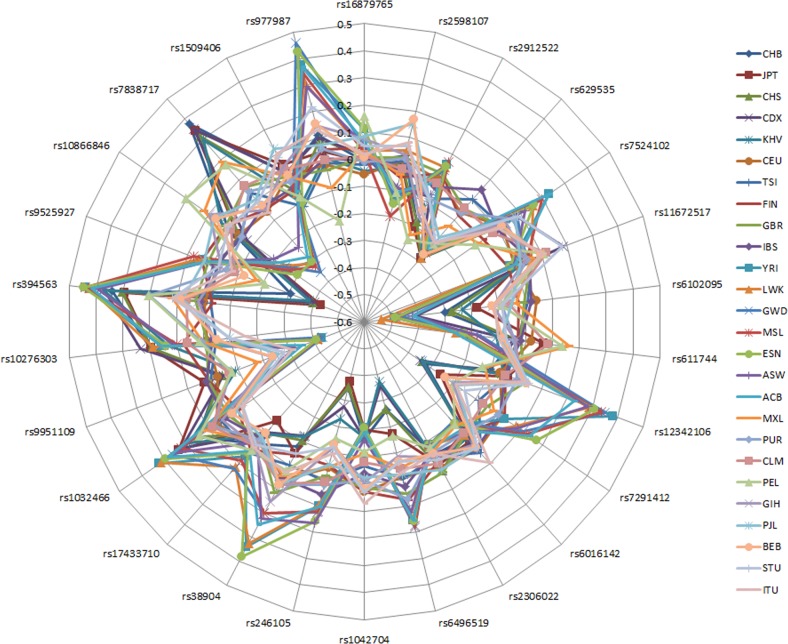
Difference in risk allele frequencies (RAFs) of the 26 DD susceptibility variants for the 26 1000 Genomes populations compared to risk allele frequencies of British GWAS controls. The SNPs are in order of highest to lowest odds ratio (clockwise starting at top). A list of the abbreviation and descriptions of the populations can be found in the supplementary data

RAFs for the 26 known variants were available from the GWAS performed by Ng et al. [26]. The details are presented in Supplementary Table 1. Those for all 1000 Genomes [28] populations in comparison to the RAFs of British GWAS controls are presented in Fig. 1 and Supplementary Table 2. Within each super-population the RAFs appear to be quite similar, but between the super-populations RAFs can be quite different. For rs629535 and rs6102095, a remarkably lower RAF was observed in non-European populations than for the British GWAS controls.

### Correlation between GRS and observed DD prevalences

The wGRSs of the 26 DD SNPs for each population were calculated and are presented in Supplementary Table 3. The uGRSs were calculated for the 26 DD SNPs and for 10,000 sets of 26 random SNPs (data for the random sets not shown), as the wGRSs cannot be calculated for random SNPs because they have not been associated with DD and hence would have no weight.

Observed prevalences of DD were determined from literature. Several reviews of DD prevalence exist, discussing populations from Europe, North-America, Asia, and Australia [1, 17]. Lanting et al. also assessed the quality of 212 studies [1]. Hereafter, several other studies on prevalence of DD in several populations were published. Below, we mention only the literature relevant to the comparison to be made with the populations from 1000 Genomes [28].

#### Europeans

Numerous studies have been carried out in Great Britain [1, 17]. Many studies were carried out in populations affected by diseases associated to DD and were therefore not considered. Several studies described the DD prevalence in the general (non-comorbid) UK population with ages ranging from 18 to 100 years: Eadington et al. described a prevalence of 18% in the general population (>40 years of age) [7], Noble et al. of 8.0% and 18.0% in two populations [3, 4], Pal et al. of 9.0% [5], Arafa et al. of 16.0% [6], and Lennox et al. of 30.0% [2]. The age-adjusted prevalences were 16.1% (Eadington et al.), 5.0% and 16.1% (Noble et al.), 9.2% (Pal et al.), and 8.1% (Lennox et al.). Arafa et al. did not specify the age range and hence prevalence was not adjusted. The mean of the prevalences weighted by their sample size was 13.4%. One epidemiological study in Italy described a DD prevalence of 3.5% in the general population of which no age range was mentioned [33]. Two studies in Finland were performed in patients with diabetes (DD prevalence in type 1 diabetics: 4%, in type 2 diabetics: 14%) and were therefore excluded [34, 35]. However, there were several studies in other Scandinavian countries, which were used instead. Mikkelson et al. described a DD prevalence of 5.6%, Bergenudd et al. of 6%, Gudmundsson et al. of 13.3%, Finsen et al. of 7.5%, and Godtfredsen of 11% [8–10, 36, 37]. One study in Spain found a DD prevalence of 8.7% (age-adjusted 7.6%) [38].

#### Africans

Several cases of DD are described in African individuals [21–23], but literature on the prevalence of DD in African populations is lacking. In 1957, Walters et al. described high DD prevalences in some Nigerian subpopulations (5.4%), but low prevalences in others (0.1%) [39]. As no age range was provided, the prevalences were not adjusted for age. The weighted prevalence for Nigerians was 4.5%. Saboeiro et al. described a 10-year retrospective study using data from Department of Veterans Affairs medical centers and estimated the DD prevalence in Americans of African descent to be 0.13% (age-adjusted 0.10%) [16]. Weinstein et al. found a prevalence of 0.29% of DD in African Americans (which was not age-adjusted because age range lacked) [20].

#### Asians

Yeh et al. described a DD prevalence of 5.7/10^5^ for 681 men and 3.4/10^5^ for 397 women (weighted mean of 4.5/10^5^) in ethnic Chinese in Taiwan (no age-adjustment applied as age range was missing) [18]. In Japanese populations, two studies on DD prevalence were carried out. Egawa et al. described a prevalence of 1.8% (age-adjusted 1.7%) [40], and Tajika et al. of 7.0% (age-adjusted 5.3%) [41]. The mean prevalence weighted by sample size for Japanese was 2.0%. Dasgupta et al. described a prevalence of 8.57% in 35 study controls of Indian descent, however, we deemed the sample size too small to be an accurate representation [42]. Srivastava et al. reported a series of 10 cases of Indian individuals living in the UK [43], however, a prevalence for this population was not available. One case of DD in a Vietnamese patient has been reported [44].

#### Hispanics

DD prevalences of 0.24% (age-adjusted 0.18%) and 0.53% (age-adjusted 0.28%) were found in Hispanic populations by Saboeiro et al. [16] and Weinstein et al. [20], respectively. The weighted mean of these age-adjusted prevalences was 0.25%.

#### Caucasian Americans

Saboeiro et al. [16] and Weinstein et al. [20] described DD prevalences of 0.73% (age-adjusted 0.55%) and 0.3% (not adjusted for age as age range was not given), respectively, in Americans from European descent. The mean weighted prevalence was 0.53%.

**Fig. 2:**
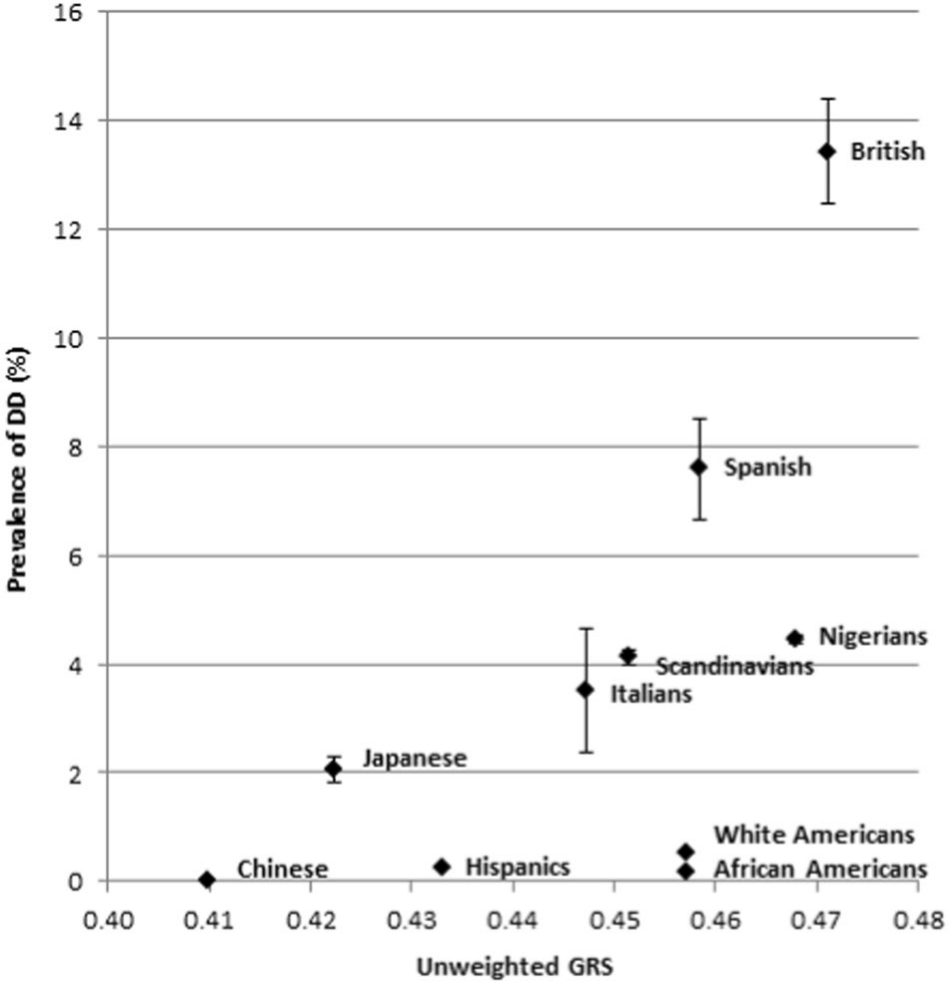
Mean unweighted GRS (*x*-axis) plotted against sample size-weighted mean of age-adjusted DD prevalences (*y*-axis) per population. Error bars represent the standard error of the prevalence estimate

Because of the type of data on DD prevalence available from literature, we chose to group some of the populations from the 1000 Genomes database [28] together. The Han Chinese in Beijing, the Southern Han Chinese, and the Chinese Dai in Xishuangbanna were grouped together as Chinese, because available literature on prevalences only provides data on Chinese in general. The same was done for Nigerians (Yoruba and Esan). When grouped, the new population’s GRS was defined as the sample size weighted mean of the GRS’ of the subpopulations concerned. The mean unweighted GRS is plotted against age-adjusted DD prevalence for each population in Fig. 2.

The correlation of unweighted GRS of the 26 DD SNPs and DD prevalences proved to be 0.60, meaning that it can explain 36% of the variance in DD prevalence between populations. The correlations between the mean GRSs and DD prevalence in the 10,000 random sets ranged from −0.80 to 0.83. Only three of the sets of 26 random SNPs showed a higher correlation with observed DD prevalence than the DD SNPs, meaning that the GRS composed of the 26 DD SNPs was significantly associated with DD prevalence (*p* = 0.0003).

### Population differentiation

**Fig. 3:**
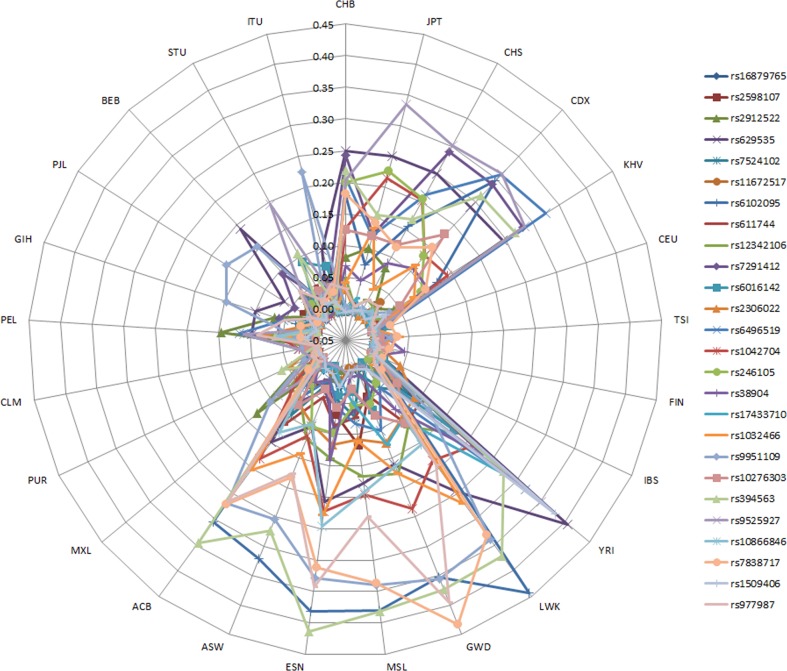
Fst values between the GBR and other populations for each DD SNP. The SNPs are in order of highest to lowest odds ratio (top to bottom). A list of the abbreviation and descriptions of the populations can be found in the supplementary data

Population Fst values for individual DD SNPs between GBR and other populations are given in Fig. 3 and Supplementary Table 4. The global Fst values per SNP ranged from 0.003 for rs11672517 to 0.151 for rs394563 and was >0.05 for 13 out of 26 SNPs. None of the individual SNP Fst values was significantly different from the Fst values from 10,000 random SNPs with similar allele frequencies SNP rs6102095 showed the highest between-population Fst (0.443 for British vs. Kenyans), but this was not significant.

Mean Fst values for all populations compared to the British were calculated using the 26 known disease variants as a set (Table 1). The population differentiation of the 26 DD variants was largest between British and Nigerians and smallest between British and Americans from European ancestry. From the super-populations, Europeans are genetically very similar to British, as expected. Interestingly, ad mixed American populations also have a similar genetic make-up of the 26 risk variants for DD. South-Asians are genetically more different, followed by the East-Asians. Africans show the largest differentiation from British.

**Table 1:** Between-population Fst values for 26 DD and random SNPs when compared to British population from 1000 Genomes (GBR)

Super-population	Population	Mean Fst of 26 DD SNPs	Mean Fst of random SNPs (range)
European	CEU	0.0027	0.0003 (−0.0037 to 0.0100)
	TSI	0.0054	0.0037 (−0.0026 to 0.0179)
	FIN	0.0051	0.0066 (−0.0020 to 0.0238)
	IBS	0.0035	0.0024 (−0.0031 to 0.0151)
Ad mixed American	MXL	0.0257	0.0328 (0.0065–0.0795)
	PUR	0.0053	0.0100 (−0.0005 to 0.0268)
	CLM	0.0037^a^	0.0149 (0.0018–0.0380)
	PEL	0.0524	0.0718 (0.0123–0.1511)
South Asian	GIH	0.0218	0.0311 (0.0045–0.0697)
	PJL	0.0155	0.0263 (0.0046–0.0560)
	BEB	0.0289	0.0350 (0.0060–0.0738)
	STU	0.0251	0.0367 (0.0067–0.0842)
	ITU	0.0287	0.0362 (0.0086–0.0834)
East Asian	CHB	0.0849	0.0745 (0.0258–0.1559)
	JPT	0.0797	0.0771 (0.0219–0.1426)
	CHS	0.0870	0.0757 (0.0203–0.1505)
	CDX	0.1019	0.0763 (0.0278–0.1501)
	KHV	0.0852	0.0736 (0.0248–0.1374)
African	YRI	0.1375^a^	0.0844 (0.0338–0.1723)
	LWK	0.1271^a^	0.0789 (0.0293–0.1966)
	GWD	0.1356^a^	0.0826 (0.0316–0.1737)
	MSL	0.1228^a^	0.0857 (0.0287–0.1703)
	ESN	0.1425^a^	0.0854 (0.0292–0.1618)
	ASW	0.0830^a^	0.0557 (0.0130–0.1222)
	ACB	0.1078^a^	0.0679 (0.0226–0.1351)

^a^Significant

The observed mean Fst values were next compared to the 10,000 random mean Fst values to assess whether populations were more or less similar to each other for the 26 DD SNPs than expected. Only one out of 25 population (CLM) had a significantly lower Fst for the 26 DD SNPs than for the sets of 26 random SNPs, implying that Colombians are more similar to the British for the 26 DD SNPs than expected. All African populations showed a larger Fst value for the 26 DD SNPs than expected from the 10,000 sets of random SNPs. Other populations were not more similar or differentiated from the British for the DD SNPs than for random SNPs.

## Discussion

In the present study, we showed that genetics could partly explain the largely differing prevalences of DD among people of different ethnic backgrounds. The correlation between observed DD prevalences from literature and mean unweighted GRS calculated from the 26 known DD SNPs was substantial (0.60), suggesting that these 26 SNPs explain 36% of variance in DD prevalence. Only three of the 10,000 correlations between DD prevalence and the mean of GRSs composed of sets of 26 random SNPs were higher than the observed correlation, meaning that the GRS composed of DD SNPs has a significant effect on DD prevalence (*p* = 0.0003). When Fst values calculated using the 26 DD SNPs were compared to Fst values calculated from 10,000 sets of random SNPs, we observed that African populations were more differentiated from the British population for the set of 26 DD SNPs than expected, while Colombians were less differentiated.

It is difficult to determine where DD first occurred historically. McFarlane [45] postulated that a genetic variation that caused DD likely occurred between 1200BC and 200BC when the age of migrations began. He argued that DD must have originated earlier than the Vikings, since DD has spread so widely by migration that it is absent in only few populations today. However, making an accurate prediction about when DD arose and whereto it has migrated is difficult, as DD is a multifactorial disease and no single genetic variant can explain this disorder. Here, we found a high prevalence of DD in European populations. Moreover, we found a substantial correlation between population prevalences and known genetic risk factors for DD, suggesting that dispersion of DD genetic risk factors due to population differentiation at least partly explains the differing prevalences of DD.

Prevalence studies on DD proved to be quite common in the European populations, particularly the British. In 2014 Lanting et al. assessed the quality of 212 prevalence studies on DD, concluding that only 23 studies had sufficient quality. Seven of these described solely male populations. Since we set out to study only populations available in the 1000 Genomes database [28], the quality assessed studies remaining for this research were of British populations only. Consequently, a downside of this research is the absence of quality assessment of prevalence studies in ethnicities other than the British. Moreover, other populations have been studied less frequently. Some research has been done in East-Asians, but almost no data exist for Hispanic, South-Asian, and African populations. Of Vietnamese, Indians from the USA, Indians from the UK, and Africans, only a handful of case reports exist [21–23, 43, 44]. Thus, unfortunately, observed prevalences of DD were not available for all 26 1000 Genomes populations [28] for correlating the GRS with DD prevalence. It would in particular be interesting to know the DD prevalence in ad-mixed American population from Central and South America, since these populations seemed similar to the British and Europeans with respect to the RAFs and Fsts for the 26 DD variants. The same holds for African populations, who were more differentiated from the British people for the DD variants than expected. Lack of information could be either due to the underreporting of DD, or because DD is rare in those populations. Although age was often reported in the prevalence studies examined, several studies were composed of patients of a limited range of age. As DD prevalence is known to increase with age, we adjusted DD prevalence for the age range of studies and used age-adjusted prevalence estimates in the analyses [1].

The etiology of DD is not still fully understood. The contribution of genetic risk in DD is estimated to be 80% in Caucasians [24], but the 26 known genetic variants account for 11.3% of variance. We calculated GRS based on the allele frequencies of those 26 SNPs and found a substantial correlation with observed DD prevalences from literature. Differences in allele frequencies of the 26 known DD SNPs between populations therefore explain for a large part the differences in DD prevalences. Risk variants other than the 26 associated with DD in Caucasians likely also play a role in the disease mechanism of DD in non-Caucasians, either different SNPs in the same genes as in the Caucasians, or SNPs in other genes. It is likely that a SNP associated in the GWAS with DD [26] is not causally involved itself but that it is in linkage disequilibrium (LD) with the disease-causing variant. LD structures surrounding this disease variant could be different in the different populations and the disease causing variant might be in LD with a DD-associated SNP in the Caucasians, but not in other ethnic groups. This could cause lower GRS values in non-Europeans and consequently bias the correlation of GRS with DD prevalence. Moreover, the differing prevalences may also stem from DD SNPs (or the variants in LD with them) having a non-additive effect, because of interactions between variants at the same locus (dominance genetic variance), or interactions between variants at different loci (epistatic genetic variance) [46]. Future research into DD heritability or finding genes for DD should focus more on non-Caucasian populations to investigate these hypotheses. The power to find disease variants in populations with smaller DD samples can be increased by weighting candidates by their evidence of natural selection [47].

Fixation indices (Fst) can be calculated for individual disease-associated SNP or for sets of SNPs. First we calculated Fst values per individual DD SNP and found global Fst >0.05 for 13 out of 26 SNPs (an Fst <0.05 is considered low implying little differentiation). SNP rs6102095 even showed an Fst as high as 0.443 between the British and Kenyan populations, but this was not significant. Secondly, we also calculated the mean Fst of the set of 26 known risk variants between the British and other populations. We showed that genotypes on the 26 known DD SNPs of the British individuals did not differ from those of the Europeans, South-Asians, and ad mixed Americans, except for the Colombian population, who was more similar to the British population than expected based on the 10,000 sets of random SNPs. Colombia is known to have a high regional diversity in ancestry, which may partly explain this finding: the Colombian population in Medellín is one known to have a strong Basque minority, and might therefore not be representative of the Colombian population as a whole. [48].

Fst values between the British and the East-Asians were moderate, and for all African populations compared to British, the Fsts were larger than expected. This is not completely in line with the findings by Myles et al. [27] and Lohmueller et al. [49], who found that despite large individual differences in allele frequencies across populations per disease-associated SNP there was no evidence for more differentiation between populations than for random SNPs. It is known that common risk alleles identified in one population may not be common in other populations. Some SNPs associated with common disease are highly differentiated in frequency across populations, because of random drift or natural selection [49]. It nevertheless remains unclear how often differences in RAFs between populations are due to local positive selection. More research in non-Caucasian populations is needed to investigate these hypotheses.

In conclusion, allele frequencies of the 26 risk variants diverged substantially between ethnic populations and provided evidence that differing DD prevalences can partly be explained by genetic differences between the populations. Understanding the mechanism behind the ethnic diversity of DD will help in scrutinizing its epidemiology and consequently will facilitate prediction of disease progression and recurrence, enabling customized care by optimizing prognostication.

## Supplementary information

Supplementary file
