# Supplementary material for: Ethnic differences in prevalence of Dupuytren disease can partly be explained by known genetic risk variants
Source: Eur J Hum Genet. 2019 Jul 30;27(12):1876–84. doi: 10.1038/s41431-019-0483-5 (PMC6871523; doi:10.1038/s41431-019-0483-5)
Supplement: Supplementary file 1 — Supplementary file [file 41431_2019_483_MOESM1_ESM.docx]

**List of abbreviations of the subpopulations**

CHB Han Chinese in Beijing, China

JPT Japanese in Tokyo, Japan

CHS Southern Han Chinese

CDX Chinese Dai in Xishuangbanna, China

KHV Kinh in Ho Chi Minh City, Vietnam

CEU Utah Residents (CEPH) with Northern and Western European Ancestry

TSI Toscani in Italia

FIN Finnish in Finland

GBR British in England and Scotland

IBS Iberian Population in Spain

YRI Yoruba in Ibadan, Nigeria

LWK Luhya in Webuye, Kenya

GWD Gambian in Western Divisions in the Gambia

MSL Mende in Sierra Leone

ESN Esan in Nigeria

ASW Americans of African Ancestry in SW USA

ACB African Caribbeans in Barbados

MXL Mexican Ancestry from Los Angeles USA

PUR Puerto Ricans from Puerto Rico

CLM Colombians from Medellin, Colombia

PEL Peruvians from Lima, Peru

GIH Gujarati Indian from Houston, Texas

PJL Punjabi from Lahore, Pakistan

BEB Bengali from Bangladesh

STU Sri Lankan Tamil from the UK

ITU Indian Telugu from the UK

**Supplementary Table 1.** SNPs Significantly Associated with Dupuytren Disease

| **Variant description** | **dbSNP ID** | **Risk Allele** | **RAF GWAS cases** | **RAF**  **GWAS controls*** | **p-value GWAS** | **OR (95% CI)** |
| --- | --- | --- | --- | --- | --- | --- |
| Hg19 chr7:37989095C>T | rs16879765 | T | 0.178 | 0.103 | 7.15 × 10^−41^ | 1.88 (1.76-2.00) |
| Hg19 chr7:37973014C>T | rs2598107 | T | 0.447 | 0.354 | 1.11 × 10^−30^ | 1.48 (1.40-1.56) |
| Hg19 chr8:69992380G>A | rs2912522 | A | 0.799 | 0.745 | 1.29 × 10^−16^ | 1.36 (1.29-1.43) |
| Hg19 chr8:70007938C>T | rs629535 | T | 0.351 | 0.285 | 2.84 × 10^−28^ | 1.36 (1.30-1.42) |
| Hg19 chr1:22698447A>G | rs7524102 | G | 0.214 | 0.168 | 7.68 × 10^−12^ | 1.35 (1.25-1.46) |
| Hg19 chr19:57678194G>A | rs11672517 | A | 0.284 | 0.228 | 1.42 × 10^−13^ | 1.34 (1.25-1.44) |
| Hg19 chr20:39320751G>A | rs6102095 | G | 0.875 | 0.840 | 8.54 × 10^−7^ | 1.34 (1.25-1.43) |
| Hg19 chr8:109228008A>G | rs611744 | A | 0.598 | 0.534 | 3.70 × 10^−19^ | 1.30 (1.24-1.36) |
| Hg19 chr9:1201156G>A | rs12342106 | A | 0.308 | 0.257 | 9.76 × 10^−12^ | 1.29 (1.23-1.35) |
| Hg19 chr22:46459132G>T | rs7291412 | T | 0.413 | 0.353 | 1.24 × 10^−15^ | 1.29 (1.24-1.35) |
| Hg19 chr20:38300807C>T | rs6016142 | T | 0.132 | 0.106 | 1.19 × 10^−6^ | 1.28 (1.20-1.36) |
| Hg19 chr15:68628163C>T | rs2306022 | T | 0.110 | 0.088 | 7.59 × 10^−6^ | 1.28 (1.19-1.37) |
| Hg19 chr15:89238184C>T | rs6496519 | C | 0.836 | 0.801 | 9.35 × 10^−8^ | 1.26 (1.20-1.34) |
| Hg19 chr14:23312594G>A | rs1042704 | A | 0.248 | 0.208 | 8.72 × 10^−13^ | 1.26 (1.20-1.32) |
| Hg19 chr5:108672946C>T | rs246105 | C | 0.799 | 0.760** | 1.34 × 10^−8^ | 1.258*** |
| Hg19 chr7:116892846T>C | rs38904 | C | 0.464 | 0.409 | 1.02 × 10^−11^ | 1.25 (1.20-1.31) |
| Hg19 chr1:162672011T>C | rs17433710 | T | 0.880 | 0.856 | 9.13 × 10^−7^ | 1.23 (1.15-1.31) |
| Hg19 chr14:51074461A>C | rs1032466 | A | 0.694 | 0.650 | 4.90 × 10^−9^ | 1.22 (1.17-1.28) |
| Hg19 chr18:9762933T>C | rs9951109 | T | 0.867 | 0.842 | 1.24 × 10^−7^ | 1.22 (1.15-1.30) |
| Hg19 chr7:3318658C>T | rs10276303 | C | 0.740 | 0.701 | 2.89 × 10^−8^ | 1.21 (1.16-1.27) |
| Hg19 chr6:149797014T>G | rs394563 | G | 0.589 | 0.543 | 2.03 × 10^−8^ | 1.21 (1.16-1.26) |
| Hg19 chr13:44842503G>A | rs9525927 | A | 0.833 | 0.806 | 5.80 × 10^−6^ | 1.20 (1.14-1.27) |
| Hg19 chr8:25845675G>A | rs10866846 | A | 0.421 | 0.379 | 3.14 × 10^−11^ | 1.19 (1.14-1.24) |
| Hg19 chr8:145504343T>C | rs7838717 | T | 0.405 | 0.365 | 2.55 × 10^−6^ | 1.18 (1.13-1.23) |
| Hg19 chr15:56229760A>G | rs1509406 | G | 0.356 | 0.322 | 4.03 × 10^−6^ | 1.16 (1.11-1.22) |
| Hg19 chr16:75506593G>A | rs977987 | G | 0.403 | 0.371 | 6.24 × 10^−7^ | 1.15 (1.10-1.20) |

Genomic reference sequence: hg19.

RAF, risk allele frequency; GWAS, genome-wide association study; OR, odds ratio; CI, confidence interval.

*RAF GWAS controls was calculated by dividing the RAF of each SNP by its OR.

** Determined from the GBR population from 1000Genomes.

*** Calculated with the RAF of the GBR population from 1000Genomes.

**Supplementary table 2.** Allele frequencies for the 26 known risk variants for all 1000Genomes populations in comparison to the risk allele frequency (RAF) of British GWAS controls.

|  | East-Asians | | | | | Europeans | | | | Africans | | | | | | | Ad-mixed Americans | | | | South-Asians | | | | |
| --- | --- | --- | --- | --- | --- | --- | --- | --- | --- | --- | --- | --- | --- | --- | --- | --- | --- | --- | --- | --- | --- | --- | --- | --- | --- |
|  | **CHB** | **JPT** | **CHS** | **CDX** | **KHV** | **CEU** | **TSI** | **FIN** | **IBS** | **YRI** | **LWK** | **GWD** | **MSL** | **ESN** | **ASW** | **ACB** | **MXL** | **PUR** | **CLM** | **PEL** | **GIH** | **PJL** | **BEB** | **STU** | **ITU** |
| **rs16879765** | -0.01 | -0.01 | 0.02 | 0.01 | -0.04 | -0.06 | -0.02 | 0.04 | 0.01 | -0.01 | 0.11 | 0.03 | 0.05 | 0.03 | 0.11 | 0.05 | 0.06 | 0.03 | -0.01 | 0.01 | 0.16 | 0.08 | 0.08 | 0.01 | 0.05 |
| **rs2598107** | 0.03 | -0.01 | -0.01 | -0.09 | -0.01 | 0.01 | 0.01 | -0.05 | 0.03 | -0.02 | -0.14 | 0.05 | -0.12 | -0.20 | -0.15 | -0.09 | -0.11 | -0.04 | 0.02 | -0.02 | -0.11 | 0.03 | 0.15 | 0.17 | 0.07 |
| **rs2912522** | -0.18 | -0.21 | -0.18 | -0.08 | -0.12 | -0.01 | -0.09 | 0.02 | 0.02 | -0.04 | 0.06 | 0.04 | 0.03 | 0.07 | 0.05 | -0.07 | -0.02 | -0.24 | -0.09 | -0.02 | -0.26 | -0.16 | -0.08 | -0.13 | -0.09 |
| **rs629535** | -0.28 | -0.28 | -0.28 | -0.28 | -0.28 | -0.06 | 0.00 | -0.08 | -0.04 | 0.05 | -0.25 | -0.28 | -0.25 | -0.26 | -0.27 | -0.22 | -0.24 | -0.13 | -0.06 | -0.04 | -0.21 | -0.21 | -0.19 | -0.27 | -0.21 |
| **rs7524102** | 0.07 | 0.08 | 0.03 | 0.08 | 0.07 | 0.02 | 0.02 | -0.07 | 0.02 | 0.00 | 0.23 | 0.16 | 0.08 | 0.20 | 0.16 | 0.12 | 0.23 | -0.09 | 0.07 | -0.02 | -0.10 | 0.04 | 0.08 | 0.02 | 0.09 |
| **rs11672517** | 0.04 | 0.02 | 0.05 | 0.19 | 0.04 | -0.01 | 0.06 | -0.03 | 0.06 | 0.05 | -0.02 | 0.06 | 0.04 | 0.04 | -0.03 | -0.01 | 0.00 | 0.06 | 0.04 | 0.12 | 0.11 | 0.03 | 0.02 | 0.10 | 0.18 |
| **rs6102095** | -0.30 | -0.18 | -0.28 | -0.41 | -0.24 | 0.04 | 0.01 | -0.03 | 0.00 | 0.02 | -0.49 | -0.54 | -0.46 | -0.48 | -0.49 | -0.42 | -0.42 | -0.03 | -0.09 | -0.06 | -0.09 | -0.12 | -0.05 | -0.12 | -0.09 |
| **rs611744** | -0.04 | 0.07 | -0.02 | -0.05 | -0.11 | 0.02 | -0.03 | -0.03 | -0.02 | -0.02 | -0.24 | -0.26 | -0.17 | -0.22 | -0.23 | -0.18 | -0.26 | 0.16 | -0.04 | 0.08 | 0.14 | -0.04 | -0.09 | -0.09 | -0.07 |
| **rs12342106** | -0.10 | -0.03 | -0.07 | -0.06 | -0.05 | -0.06 | -0.04 | -0.04 | 0.03 | -0.05 | 0.38 | 0.29 | 0.35 | 0.34 | 0.31 | 0.28 | 0.23 | -0.12 | 0.00 | -0.04 | -0.13 | 0.02 | 0.03 | 0.04 | 0.05 |
| **rs7291412** | -0.32 | -0.26 | -0.34 | -0.35 | -0.34 | 0.02 | -0.09 | 0.03 | -0.05 | 0.01 | 0.03 | 0.08 | 0.11 | 0.13 | 0.17 | 0.09 | 0.14 | -0.02 | 0.00 | -0.07 | -0.20 | -0.20 | -0.19 | -0.24 | -0.15 |
| **rs6016142** | -0.03 | -0.01 | -0.05 | -0.01 | -0.03 | 0.03 | 0.05 | -0.05 | -0.04 | 0.02 | -0.09 | -0.04 | -0.11 | -0.09 | -0.10 | -0.05 | -0.06 | -0.09 | -0.05 | -0.05 | -0.09 | 0.01 | 0.02 | 0.02 | 0.03 |
| **rs2306022** | -0.04 | -0.01 | 0.02 | 0.02 | -0.01 | 0.01 | -0.04 | -0.02 | 0.03 | -0.06 | -0.09 | -0.08 | -0.09 | -0.09 | -0.09 | -0.09 | -0.07 | -0.06 | -0.05 | -0.01 | -0.08 | -0.03 | 0.02 | -0.05 | -0.02 |
| **rs6496519** | -0.26 | -0.17 | -0.27 | -0.36 | -0.37 | -0.01 | 0.00 | 0.08 | 0.06 | 0.03 | 0.16 | 0.15 | 0.18 | 0.18 | 0.16 | 0.12 | 0.16 | -0.04 | 0.07 | -0.04 | -0.17 | -0.09 | -0.02 | -0.07 | -0.07 |
| **rs1042704** | -0.16 | -0.20 | -0.20 | -0.17 | -0.17 | 0.03 | -0.07 | 0.03 | 0.01 | -0.04 | -0.21 | -0.19 | -0.21 | -0.20 | -0.21 | -0.17 | -0.19 | -0.11 | -0.02 | -0.08 | -0.12 | -0.01 | -0.03 | 0.02 | 0.03 |
| **rs246105** | -0.35 | -0.37 | -0.35 | -0.28 | -0.23 | -0.04 | 0.01 | -0.05 | -0.01 | 0.06 | 0.10 | 0.10 | 0.13 | 0.12 | 0.16 | 0.17 | 0.10 | -0.05 | 0.02 | 0.01 | -0.15 | -0.12 | -0.06 | -0.13 | -0.12 |
| **rs38904** | -0.08 | -0.05 | -0.10 | -0.12 | -0.12 | 0.06 | 0.00 | -0.04 | 0.11 | 0.05 | 0.34 | 0.32 | 0.20 | 0.20 | 0.38 | 0.22 | 0.25 | 0.04 | 0.08 | 0.08 | 0.02 | 0.15 | 0.08 | 0.08 | -0.05 |
| **rs17433710** | 0.00 | -0.11 | -0.05 | -0.06 | -0.04 | 0.02 | 0.05 | 0.03 | -0.02 | -0.02 | 0.06 | 0.12 | 0.13 | 0.08 | 0.05 | 0.00 | 0.04 | 0.00 | -0.02 | 0.01 | 0.03 | 0.01 | -0.03 | -0.05 | 0.01 |
| **rs1032466** | 0.15 | 0.23 | 0.15 | 0.22 | 0.19 | 0.06 | 0.04 | -0.03 | 0.01 | 0.03 | 0.32 | 0.31 | 0.26 | 0.22 | 0.29 | 0.25 | 0.28 | 0.11 | 0.08 | 0.08 | 0.14 | 0.04 | -0.02 | 0.00 | -0.05 |
| **rs9951109** | -0.03 | 0.03 | -0.04 | -0.04 | -0.09 | -0.02 | 0.01 | 0.02 | 0.02 | 0.03 | -0.43 | -0.42 | -0.44 | -0.41 | -0.41 | -0.33 | -0.35 | -0.18 | -0.07 | -0.07 | -0.08 | -0.24 | -0.26 | -0.24 | -0.28 |
| **rs10276303** | 0.20 | 0.19 | 0.19 | 0.23 | 0.13 | -0.01 | -0.05 | 0.08 | -0.01 | -0.03 | 0.16 | 0.19 | 0.16 | 0.10 | 0.14 | 0.11 | 0.16 | 0.00 | 0.01 | 0.06 | 0.02 | -0.04 | -0.10 | -0.05 | -0.10 |
| **rs394563** | 0.34 | 0.29 | 0.30 | 0.37 | 0.37 | 0.00 | -0.03 | -0.03 | 0.03 | 0.01 | 0.44 | 0.43 | 0.42 | 0.44 | 0.45 | 0.39 | 0.42 | 0.11 | 0.20 | 0.06 | 0.20 | 0.02 | 0.08 | 0.09 | 0.11 |
| **rs9525927** | -0.31 | -0.43 | -0.40 | -0.40 | -0.38 | -0.02 | -0.02 | 0.04 | 0.02 | -0.01 | 0.04 | 0.05 | 0.03 | 0.08 | 0.02 | 0.02 | 0.03 | -0.17 | -0.06 | -0.09 | -0.21 | -0.03 | 0.03 | -0.13 | -0.02 |
| **rs10866846** | 0.01 | -0.03 | -0.03 | -0.07 | 0.04 | -0.04 | -0.04 | 0.02 | 0.02 | -0.05 | -0.25 | -0.24 | -0.24 | -0.27 | -0.30 | -0.19 | -0.22 | 0.12 | -0.05 | 0.00 | 0.20 | 0.10 | 0.07 | 0.07 | 0.01 |
| **rs7838717** | 0.38 | 0.34 | 0.32 | 0.35 | 0.30 | -0.06 | 0.03 | -0.05 | 0.06 | -0.03 | -0.31 | -0.31 | -0.36 | -0.32 | -0.30 | -0.23 | -0.28 | 0.19 | 0.04 | 0.07 | 0.18 | -0.05 | 0.00 | -0.03 | -0.01 |
| **rs1509406** | 0.02 | 0.05 | -0.06 | 0.02 | -0.10 | 0.01 | -0.12 | -0.05 | 0.01 | -0.04 | -0.07 | -0.06 | -0.03 | -0.03 | -0.10 | -0.07 | -0.06 | 0.01 | -0.01 | 0.03 | -0.08 | 0.05 | 0.12 | 0.01 | 0.08 |
| **rs977987** | 0.11 | 0.05 | 0.09 | 0.10 | 0.02 | 0.00 | 0.00 | 0.05 | -0.02 | 0.02 | 0.37 | 0.31 | 0.46 | 0.34 | 0.43 | 0.29 | 0.37 | -0.09 | 0.06 | 0.01 | -0.22 | 0.14 | 0.07 | 0.15 | 0.22 |

**Supplementary Table 3.** Unweighted and weighted genetic risk scores (GRS and wGRS, respectively) of the 26 DD SNPs for each population from 1000Genomes

**.**

|  |  |  |  |  |
| --- | --- | --- | --- | --- |
|  |  |  |  |  |
|  |  |  |  |  |
|  |  |  |  |  |
|  |  |  |  |  |
|  |  |  |  |  |
|  |  |  |  |  |
|  |  |  |  |  |
|  |  |  |  |  |
|  |  |  |  |  |
|  |  |  |  |  |
|  |  |  |  |  |
|  |  |  |  |  |
|  |  |  |  |  |
|  |  |  |  |  |
|  |  |  |  |  |
|  |  |  |  |  |
|  |  |  |  |  |
|  |  |  |  |  |
|  |  |  |  |  |
|  |  |  |  |  |
|  |  |  |  |  |
|  |  |  |  |  |
|  |  |  |  |  |
|  |  |  |  |  |
|  |  |  |  |  |
|  |  |  |  |  |
|  | | | | |
|  | | |  |  |
| **Population** | | | **uGRS** | **wGRS** |
| ACB | | | 0.466 | 0.107 |
| ASW | | | 0.457 | 0.105 |
| BEB | | | 0.428 | 0.099 |
| CDX | | | 0.413 | 0.093 |
| CEU | | | 0.457 | 0.106 |
| CHB | | | 0.416 | 0.093 |
| CHS | | | 0.400 | 0.090 |
| CLM | | | 0.461 | 0.107 |
| ESN | | | 0.468 | 0.108 |
| FIN | | | 0.452 | 0.105 |
| GBR | | | 0.471 | 0.110 |
| GIH | | | 0.435 | 0.101 |
| GWD | | | 0.466 | 0.106 |
| IBS | | | 0.459 | 0.107 |
| ITU | | | 0.431 | 0.100 |
| JPT | | | 0.422 | 0.095 |
| KHV | | | 0.399 | 0.090 |
| LWK | | | 0.469 | 0.108 |
| MSL | | | 0.458 | 0.104 |
| MXL | | | 0.433 | 0.100 |
| PEL | | | 0.417 | 0.097 |
| PJL | | | 0.450 | 0.106 |
| PUR | | | 0.463 | 0.107 |
| STU | | | 0.444 | 0.103 |
| TSI | | | 0.447 | 0.104 |
| YRI | | | 0.468 | 0.108 |

uGRS, unweighted genetic risk score; wGRS, weighted genetic risk score

**Supplementary Table 4**. Fst values between the GBR and other populations for each DD SNP.

|  | East-Asians | | | | | Europeans | | | | Africans | | | | | | | Ad-mixed Americans | | | | South-Asians | | | | | **Mean fst** |
| --- | --- | --- | --- | --- | --- | --- | --- | --- | --- | --- | --- | --- | --- | --- | --- | --- | --- | --- | --- | --- | --- | --- | --- | --- | --- | --- |
|  | **CHB** | **JPT** | **CHS** | **CDX** | **KHV** | **CEU** | **TSI** | **FIN** | **IBS** | **YRI** | **LWK** | **GWD** | **MSL** | **ESN** | **ASW** | **ACB** | **MXL** | **PUR** | **CLM** | **PEL** | **GIH** | **PJL** | **BEB** | **STU** | **ITU** |  |
| **rs16879765** | 0.00 | 0.00 | -0.01 | -0.01 | 0.01 | 0.02 | 0.00 | 0.00 | 0.00 | 0.03 | 0.00 | 0.00 | 0.00 | 0.04 | 0.00 | 0.01 | 0.00 | 0.00 | -0.01 | 0.07 | 0.01 | 0.02 | -0.01 | 0.00 | 0.00 | 0.01 |
| **rs2598107** | -0.01 | 0.00 | 0.00 | 0.02 | 0.00 | 0.00 | 0.00 | 0.01 | 0.00 | 0.06 | 0.00 | 0.05 | 0.12 | 0.07 | 0.02 | 0.04 | 0.00 | 0.00 | 0.00 | 0.03 | -0.01 | 0.03 | 0.04 | 0.00 | 0.00 | 0.02 |
| **rs2912522** | 0.08 | 0.10 | 0.08 | 0.02 | 0.04 | 0.00 | 0.02 | -0.01 | 0.00 | 0.00 | 0.00 | 0.00 | 0.00 | 0.00 | 0.01 | 0.00 | 0.13 | 0.02 | 0.00 | 0.15 | 0.07 | 0.02 | 0.05 | 0.02 | 0.05 | 0.03 |
| **rs629535** | 0.19 | 0.25 | 0.25 | 0.24 | 0.24 | 0.00 | 0.00 | 0.00 | 0.02 | 0.17 | 0.24 | 0.16 | 0.18 | 0.21 | 0.10 | 0.15 | 0.01 | 0.00 | -0.01 | 0.10 | 0.10 | 0.06 | 0.19 | 0.09 | 0.10 | 0.12 |
| **rs7524102** | 0.00 | 0.00 | 0.00 | 0.01 | 0.00 | -0.01 | -0.01 | 0.02 | 0.00 | 0.10 | 0.04 | 0.01 | 0.07 | 0.05 | 0.02 | 0.09 | 0.04 | 0.00 | 0.00 | 0.06 | 0.00 | 0.00 | -0.01 | 0.01 | 0.00 | 0.02 |
| **rs11672517** | 0.00 | 0.00 | -0.01 | 0.03 | 0.00 | 0.01 | 0.00 | 0.02 | 0.00 | 0.01 | -0.01 | 0.00 | 0.00 | 0.01 | 0.00 | 0.00 | -0.01 | -0.01 | 0.00 | 0.00 | 0.00 | 0.00 | 0.00 | 0.03 | 0.00 | 0.00 |
| **rs6102095** | 0.18 | 0.07 | 0.16 | 0.30 | 0.12 | 0.00 | 0.00 | 0.00 | 0.00 | 0.38 | 0.44 | 0.35 | 0.38 | 0.38 | 0.32 | 0.30 | 0.00 | 0.02 | 0.01 | 0.02 | 0.03 | 0.00 | 0.04 | 0.02 | 0.03 | 0.14 |
| **rs611744** | 0.00 | 0.01 | 0.00 | 0.00 | 0.01 | 0.00 | 0.00 | -0.01 | 0.00 | 0.09 | 0.11 | 0.04 | 0.07 | 0.09 | 0.05 | 0.11 | 0.06 | 0.00 | 0.02 | 0.04 | 0.00 | 0.01 | 0.00 | 0.00 | 0.01 | 0.03 |
| **rs12342106** | 0.05 | 0.01 | 0.02 | 0.02 | 0.01 | 0.02 | 0.01 | 0.01 | 0.01 | 0.21 | 0.12 | 0.18 | 0.17 | 0.14 | 0.12 | 0.07 | 0.06 | 0.00 | 0.01 | 0.08 | 0.00 | -0.01 | -0.01 | -0.01 | -0.01 | 0.05 |
| **rs7291412** | 0.24 | 0.12 | 0.29 | 0.29 | 0.28 | 0.00 | 0.00 | 0.01 | 0.00 | 0.01 | 0.03 | 0.05 | 0.06 | 0.09 | 0.04 | 0.07 | 0.00 | 0.00 | -0.01 | 0.06 | 0.06 | 0.05 | 0.09 | 0.02 | 0.05 | 0.08 |
| **rs6016142** | 0.00 | 0.00 | 0.00 | 0.00 | 0.00 | 0.02 | 0.03 | 0.00 | 0.02 | 0.02 | 0.00 | 0.07 | 0.03 | 0.04 | -0.01 | 0.00 | 0.02 | 0.00 | -0.01 | 0.03 | 0.01 | 0.01 | 0.02 | 0.02 | 0.07 | 0.02 |
| **rs2306022** | 0.03 | 0.00 | 0.00 | 0.00 | 0.00 | 0.00 | 0.02 | 0.01 | 0.05 | 0.12 | 0.10 | 0.12 | 0.11 | 0.12 | 0.09 | 0.08 | 0.04 | 0.04 | 0.00 | 0.08 | 0.02 | 0.00 | 0.03 | 0.01 | 0.04 | 0.04 |
| **rs6496519** | 0.21 | 0.12 | 0.21 | 0.31 | 0.33 | 0.01 | 0.01 | 0.00 | 0.00 | 0.06 | 0.04 | 0.10 | 0.08 | 0.06 | 0.02 | 0.05 | 0.03 | 0.00 | 0.02 | 0.12 | 0.05 | 0.01 | 0.04 | 0.04 | 0.02 | 0.08 |
| **rs1042704** | 0.13 | 0.21 | 0.20 | 0.13 | 0.14 | 0.00 | 0.02 | 0.00 | 0.00 | 0.23 | 0.18 | 0.24 | 0.20 | 0.22 | 0.11 | 0.18 | 0.05 | 0.00 | 0.02 | 0.06 | 0.00 | 0.00 | -0.01 | 0.00 | 0.00 | 0.09 |
| **rs246105** | 0.20 | 0.23 | 0.20 | 0.13 | 0.09 | 0.00 | 0.00 | 0.00 | 0.01 | 0.04 | 0.03 | 0.06 | 0.05 | 0.10 | 0.10 | 0.04 | 0.00 | 0.00 | 0.00 | 0.04 | 0.02 | 0.00 | 0.03 | 0.02 | 0.01 | 0.06 |
| **rs38904** | 0.07 | 0.05 | 0.09 | 0.11 | 0.10 | 0.00 | 0.02 | 0.04 | 0.00 | 0.10 | 0.09 | 0.01 | 0.01 | 0.14 | 0.02 | 0.03 | 0.00 | 0.00 | 0.00 | 0.01 | 0.00 | 0.00 | 0.00 | 0.05 | 0.01 | 0.04 |
| **rs17433710** | 0.00 | 0.02 | 0.00 | 0.00 | 0.00 | 0.00 | 0.01 | 0.01 | -0.01 | 0.03 | 0.11 | 0.13 | 0.05 | 0.02 | -0.01 | 0.01 | -0.01 | 0.00 | 0.00 | 0.01 | 0.00 | -0.01 | 0.00 | 0.00 | 0.00 | 0.01 |
| **rs1032466** | 0.04 | 0.13 | 0.04 | 0.11 | 0.08 | 0.00 | 0.00 | 0.00 | 0.00 | 0.29 | 0.27 | 0.18 | 0.11 | 0.23 | 0.14 | 0.20 | 0.02 | 0.01 | 0.01 | 0.04 | 0.00 | 0.00 | -0.01 | 0.00 | 0.00 | 0.08 |
| **rs9951109** | 0.00 | 0.00 | 0.01 | 0.01 | 0.03 | 0.00 | 0.00 | -0.01 | 0.00 | 0.35 | 0.34 | 0.35 | 0.34 | 0.33 | 0.25 | 0.27 | 0.10 | 0.02 | 0.02 | 0.03 | 0.15 | 0.17 | 0.15 | 0.19 | 0.22 | 0.13 |
| **rs10276303** | 0.13 | 0.12 | 0.12 | 0.18 | 0.05 | -0.01 | 0.00 | 0.02 | 0.00 | 0.08 | 0.11 | 0.08 | 0.03 | 0.06 | 0.03 | 0.08 | -0.01 | 0.00 | 0.01 | 0.00 | 0.00 | 0.01 | 0.00 | 0.01 | 0.04 | 0.04 |
| **rs394563** | 0.22 | 0.15 | 0.17 | 0.26 | 0.27 | 0.00 | 0.00 | 0.00 | 0.00 | 0.41 | 0.37 | 0.37 | 0.38 | 0.41 | 0.27 | 0.35 | 0.01 | 0.06 | 0.00 | 0.06 | 0.00 | 0.00 | 0.00 | 0.01 | 0.01 | 0.15 |
| **rs9525927** | 0.20 | 0.33 | 0.30 | 0.31 | 0.29 | 0.00 | 0.00 | 0.00 | 0.00 | 0.00 | 0.00 | 0.00 | 0.01 | 0.00 | -0.01 | -0.01 | 0.09 | 0.01 | 0.03 | 0.11 | 0.00 | 0.00 | 0.05 | 0.00 | 0.02 | 0.07 |
| **rs10866846** | -0.01 | 0.00 | 0.00 | 0.01 | 0.00 | 0.00 | 0.00 | -0.01 | 0.01 | 0.18 | 0.15 | 0.16 | 0.19 | 0.25 | 0.09 | 0.13 | 0.01 | 0.01 | 0.00 | 0.06 | 0.01 | 0.00 | 0.00 | -0.01 | -0.01 | 0.05 |
| **rs7838717** | 0.18 | 0.14 | 0.12 | 0.15 | 0.10 | 0.03 | 0.00 | 0.02 | 0.01 | 0.32 | 0.33 | 0.43 | 0.34 | 0.31 | 0.18 | 0.27 | 0.03 | 0.00 | -0.01 | 0.02 | 0.02 | 0.00 | 0.01 | 0.01 | 0.03 | 0.12 |
| **rs1509406** | 0.00 | 0.00 | 0.00 | -0.01 | 0.03 | -0.01 | 0.04 | 0.00 | 0.00 | 0.01 | 0.01 | 0.00 | 0.00 | 0.03 | 0.01 | 0.00 | -0.01 | 0.00 | 0.00 | 0.01 | 0.00 | 0.02 | -0.01 | 0.01 | 0.01 | 0.01 |
| **rs977987** | 0.03 | 0.01 | 0.02 | 0.03 | 0.00 | 0.00 | 0.00 | 0.01 | 0.00 | 0.28 | 0.19 | 0.39 | 0.23 | 0.34 | 0.18 | 0.27 | 0.00 | 0.01 | 0.00 | 0.09 | 0.05 | 0.01 | 0.06 | 0.11 | 0.04 | 0.09 |
